# Draft genome sequences and pan-genome of *Bacillus* Bac5 and Bac6 strains isolated from a mangrove ecosystem with anti-Vibrio activity

**DOI:** 10.1016/j.dib.2026.113363

**Published:** 2026-10-22

**Authors:** Guillermo Reyes, Lita Sorroza

**Affiliations:** a Centro Nacional de Acuicultura e Investigaciones Marinas, CENAIM, Escuela Superior Politécnica del Litoral, ESPOL, Guayaquil, Ecuador; b Facultad de Ciencias Agropecuarias, Universidad Técnica de Machala, Machala, Ecuador

**Keywords:** Mangroves, Pacific white shrimp, Whole-genome sequencing, Illumina novaseq x plus, Virulent factor genes, Antimicrobial, Shimp-pathogenic vibrio, Bacteriocin gene cluster, Probiotics in aquaculture

## Abstract

Two *Bacillus* strains (*Bacillus velezensis* Bac5 and *Bacillus subtilis* Bac6) displaying antagonistic activity against *Vibrio* spp. were isolated from mangrove sediments in Ecuador. Here, we report the draft genome sequences and provide a comprehensive genomic characterization. The genome of strain Bac5 has a size of 3889,715 bp assembled into 15 contigs, while Bac6 has a genome size of 4384,991 bp assembled into 20 contigs. Genome annotation identified 4 and 5 virulence-associated genes, respectively, including genes related to motility, iron acquisition, capsule formation, stress response, and toxin-associated regulators commonly reported in *Bacillus* species. In addition, several antimicrobial resistance-related genes were detected. The most abundant COG functional categories in both strains were transcription, amino acid transport and metabolism, and carbohydrate transport and metabolism, indicating metabolic versatility. To further contextualize these findings, a comparative pan-genome analysis was performed, including our genomes and publicly available *B. velezensis* and *B. subtilis* strains associated with aquaculture and other applications. However, gene association analysis did not identify statistically significant markers linked to aquaculture-associated strains. Biosynthetic gene cluster (BGC) analysis revealed that both Bac5 and Bac6 harbor a conserved repertoire of antimicrobial clusters commonly reported in the species, including lipopeptides and polyketides, consistent with their antagonistic activity. In contrast, genomic differences were observed between *B. velezensis* and *B. subtilis*, reflecting species-level divergence rather than traits associated with probiotic function. The objective of this study was to sequence and characterize the genomes of two *B. velezensis* (Bac5) and *B. subtilis* (Bac6) strains with anti-Vibrio activity, and to compare them with genomes from aquaculture-associated and other environments through pangenome and biosynthetic gene cluster analyzes. This dataset enables comparative genomic analyzes of probiotic-associated features, highlighting a conserved core genome and a variable accessory gene repertoire across strains from different ecological and applied contexts.

Specifications Table

**Table:** 

Subject	Omics: Genomics
Specific subject area	Bacterial Genome: Genomics, Bioinformatics, and Phylogeny.
Type of data	Figures: Circular genome maps of *Bacillus* spp*.*, phylogenetic tree analysis. FASTA: Files containing the genomic sequence data.
Data collection	Two bacterial strains were isolated from mangrove sediments in Puerto Hualtaco, Ecuador. Genomic DNA was extracted using the Quick-DNA HMW MagBead Kit (Zymo Research, USA); DNA quality check: Dnovix DS-11 Spectrophotometer (Dnovix, USA) and 2% (w/v) agarose horizontal gel electrophoresis; Sequencing Illumina NovaSeq X plus; Assembly: Bactopia pipeline; genome identification and annotation: PGAP, NCBI; Percentage identity: BLAST nucleotide, NCBI; Multiple sequence alignment and Phylogenetic tree: TYGS.
Data source location	The bacteria were isolated from mangrove sediments collected in Puerto Hualtaco, Huaquillas, El Oro Province, Ecuador (3°26′29″ S, 80°14′54″ W).
Data accessibility	Repository name: The assembled and annotated genomes, along with the raw sequencing data (FASTQ and FASTA), for the two *Bacillus* strains have been deposited in the NCBI Genbank under the accession numbers JBWDLW000000000 (bac5) and JBWDLX000000000 (bac6). The raw reads are also available in the SRA under the BioSample accessions SAMN52959777 and SAMN52959749. Direct link to the dataset: https://www.ncbi.nlm.nih.gov/bioproject/PRJNA1354868/. https://www.ncbi.nlm.nih.gov/nuccore/JBWDLW000000000/. https://www.ncbi.nlm.nih.gov/nuccore/JBWDLX000000000.1/.
Related research article	None.

## 1 Value of the Data

• The draft genome sequences of these two *Bacillus* strains, isolated from mangrove environments, provide novel genomic resources from an underexplored niche relevant to aquaculture. These strains were selected based on their antagonistic activity against shrimp-pathogenic *Vibrio parahaemolyticus*, linking genomic features with a defined functional phenotype.
• These data help address gaps in current genomic reference datasets by incorporating strains from coastal and estuarine ecosystems, which remain underrepresented in probiotic-focused studies. They support comparative and pangenome analyzes of *Bacillus subtilis* and *Bacillus velezensis*, particularly for evaluating bacteriocin gene cluster profiles.
• The genomes are relevant for downstream applications in molecular taxonomy, phylogenomics, aquaculture biotechnology, and antimicrobial resistance (AMR) surveillance*.*
• While lactic acid bacteria (LAB) have been extensively studied as probiotics with well-established genomic frameworks for safety and functionality assessment, comparable integrative genomic analyzes in *Bacillus* spp. remain less developed. In this context, these data contribute to bridging this gap by supporting genome-informed evaluation of *Bacillus* strains, complementing existing knowledge derived from LAB-based probiotic systems.

## 2 Background

*Bacillus* spp. are widely used as probiotics in aquaculture due to their antagonistic activity against bacterial pathogens and their capacity to improve host performance and health [1,2]. In shrimp farming, infections caused by *Vibrio parahaemolyticus* remain a major concern, motivating the continued search for effective biocontrol strategies [3]. In this context, *Bacillus* strains have been applied to enhance disease resistance, growth, and immune responses in *Penaeus vannamei* [1,[4], [5], [6]]. Mangrove ecosystems represent a promising yet underexplored source of microbial diversity, where environmental pressures may select for bacteria with traits advantageous for aquaculture applications [7]. However, the selection of probiotic candidates requires careful evaluation, as even environmental isolates may harbor antimicrobial resistance or virulence-associated genes [[8], [9], [10], [11], [12]]. Genome-based comparative analyzes are therefore essential to simultaneously assess biosafety-related features and biosynthetic potential, particularly the presence of biosynthetic gene clusters (BCGs) involved in the production of antimicrobial compounds such as lipopeptides and polyketides [13,14]. Although numerous genomes of *B. subtilis* and *B. velezensis* are publicly available, only a limited subset has been characterized in the context of aquaculture probiotics using integrated pangenome and BGC approaches. This limits our understanding of the relationship between ecological origin, genomic diversity, and functional potential in these strains. In this context, pangenome analysis provides a powerful framework to identify conserved and accessory genomic features associated with both biosafety and secondary metabolite production. In this study, we characterize two *Bacillus* strains isolated from mangrove sediments with inhibitory activity against shrimp-pathogenic *Vibrio parahaemolyticus* and compare them with publicly available genomes from aquaculture and other environments to evaluate their genomic diversity, biosafety-related traits, and biosynthetic gene cluster content.

## 3 Data Description

We report the draft genomes of *Bacillus* strains Bac5 and Bac6, measuring 3889,715 bp and 4384,991 bp, respectively, and assembled into 15 and 20 contigs. Strain Bac5 comprises 3836 predicted genes, while Bac6 contains 4524 genes (Fig. 1 and Table 1). PathogenFinder2 analysis indicated very low probabilities of human pathogenicity for both strains, which means scores of 0.0436 for Bac5 and 0.0691 for Bac6. Average nucleotide identity (ANI) comparisons revealed 98.08% similarity between Bac5 and *B. velezensis* strain FZB42 (GenBank accession GCA_000015785.2), and 99.99% between Bac6 and *B. subtilis* strain 168 (GenBank accession GCA_000009045.1).

**Fig. 1:**
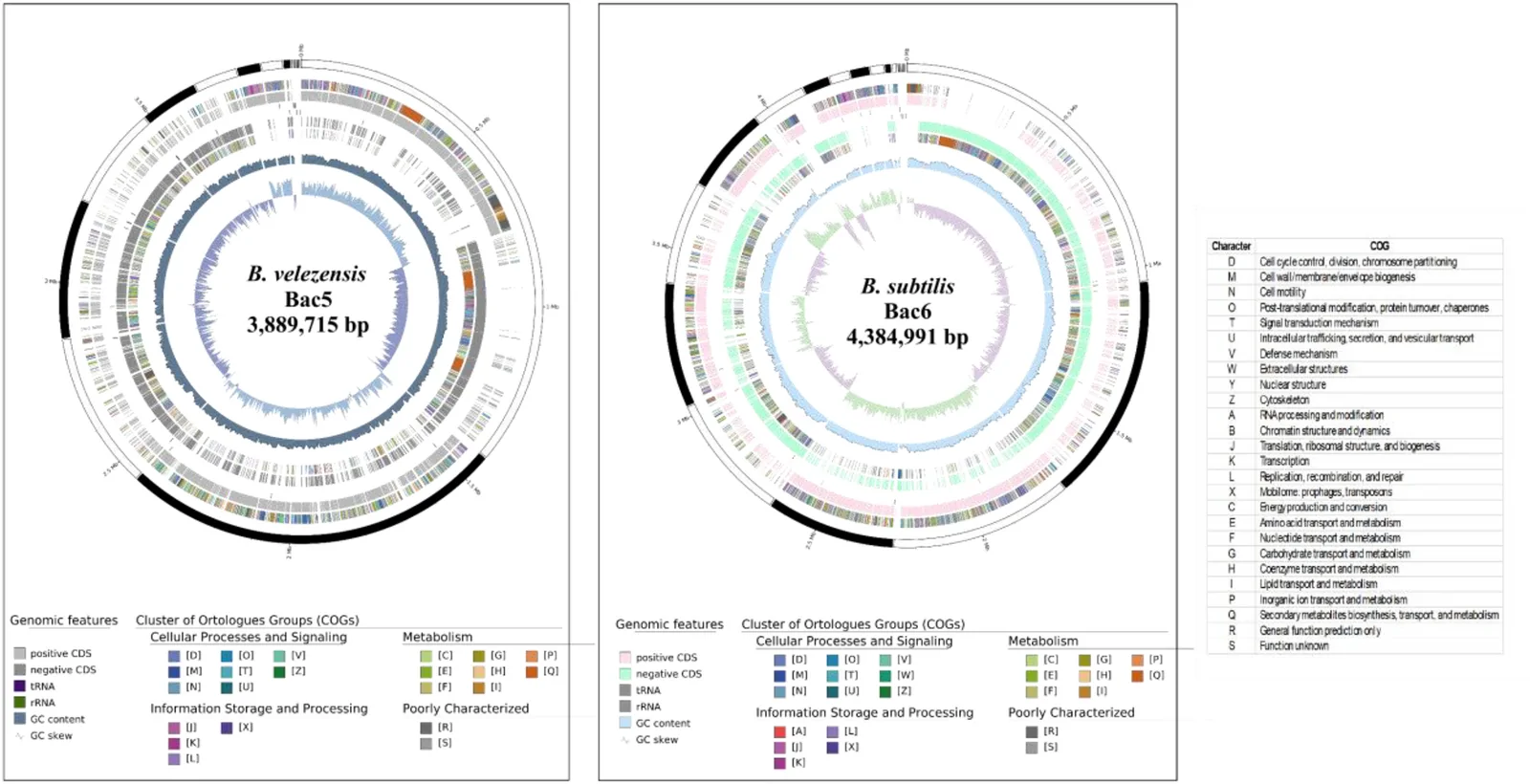
Visualization of the genomes of *Bacillus* Bac5 and Bac6. A) Circular genome map of Bac5 strain, displaying (from the center outward) GC skew, GC content, rRNA and tRNA, and coding sequences on the negative and positive strands. B) Circular genome map of Bac6 strain. Functional gene categories are assigned based on the COG database, which color codes explained in the accompanying table.

**Table 1:** Assembly metrics and annotated genomic characteristics of the two *Bacillus* strains.

Item	Value	
	Bac5	Bac6
Genome length (bp)	3889,715	4384,991
Contigs	15	20
Coverage (X)	762	488
GC content (%)	46.90	45.45
Total of genes	3836	4625
Total No of CDS	3749	4524
rRNAs	4	4
tRNAs	82	96
tmRNAs	1	1

Both strains shared genes associated with efflux-mediated resistance (e.g., *tet, qac* family) and putative glycopeptide resistance clusters, although with relatively low sequence identity (Supplementary Table 1). Table 2 summarizes the virulence-associated genes identified in the Bac5 and Bac6 strains. Bac5 contains 4 virulence genes, whereas Bac6 harbors 5 genes.

**Table 2:** Predicted virulence-associated genes in *Bacillus* Bac5 and Bac6 based on sequence similarity searches against the VFDB (≥70% identity, ≥60% coverage).

Strain	Gene	Identity (%)	Coverage (%)	Start	End	Strand	Gene function	Category
Bac5	*capC*	70.67	100.00	496,986	497,435	+	Poly-γ-glutamate synthesis	Immune modulation
Bac5	*clpP*	72.38	92.13	634,135	634,695	-	ATP-dependent protease	Stress survival
Bac5	*clpC*	70.54	95.29	2346	4703	+	ATP-binding protease	Stress survival
Bac5	*tufA*	72.48	99.32	31,638	32,828	+	Elongation factor Tu	Adherence
Bac6	*clpP*	73.49	93.47	367,503	368,062	+	ATP-dependent protease	Stress survival
Bac6	*capC*	71.17	99.78	520,276	520,725	-	Poly-γ-glutamate synthesis	Immune modulation
Bac6	*clpC*	71.68	95.01	2361	4718	+	ATP-binding protease	Stress survival
Bac6	*tufA*	72.66	99.58	31,671	32,861	+	Elongation factor Tu	Adherence
Bac6	*clpE*	70.38	64.28	557,431	558,848	+	ATP-dependent protease	Stress survival

Phaster/Phastest analysis of the Bac5 genome identified two prophage regions. The first region, approximately 12.6 Kb in length, was classified as incomplete and showed similarity to *Bacillus* phage SPβ (NC_001884). The second region, measuring 18.9 Kb, was categorized as questionable and exhibited homology to *Brevibacillus* phage Jimmer1 (NC_029104). Bac5 and Bac6 displayed distinct bacteriocin gene cluster profiles, with Bac6 exhibiting a more diverse repertoire of RiPP-associated clusters compared to Bac5, indicating strain-specific antimicrobial potential (Table 3); this variability is consistent with the pangenome analysis, which showed that bacteriocin-related clusters are part of the accessory genome and are not conserved across all strains (Supplementary Tables 2 and 3).

**Table 3:** Comparative distribution of biosynthetic gene clusters across analyzed genomes of *Bacillus velezensis* and *Bacillus subtilis.*

BGC / Metabolite	Functional category	Bac5	Bac6
**Surfactin**	Lipopeptide	Present	Present
**Fengycin**	Lipopeptide	Present	Present
**Iturin/Bacillomycin**	Lipopeptide	Partial/overlapping	Partial/overlapping
**Difficidin**	Polyketide	Present	Absent
**Bacillaene**	Polyketide	Present	Present
**Macrolactin**	Polyketide	Present	Absent
**Bacilysin**	Dipeptide antibiotic	Present	Present
**Bacillibactin**	Siderophore	Present	Present
**Pulcherriminic acid**	Iron acquisition	Absent	Present
**Subtilosin A**	RiPP (bacteriocin)	Absent	Present
**Sublancin 168**	RiPP (bacteriocin)	Absent	Present
**Amylocyclicin**	RiPP (bacteriocin)	Present	Absent
**LCI-like bacteriocin**	RiPP	Present	Absent
**Sactipeptides**	RiPP	Present	Present
**Sporulation killing factor (SKF)**	RiPP / competition	Absent	Present
**Lanthipeptide class II**	RiPP	Present	Present/variant
**Terpenes**	Secondary metabolite	Present	Present
**T3PKS (unknown function)**	Polyketide-like	Present	Present
**NRPS/PKS hybrids**	Mixed BGCs	Present	Present
**Low-similarity clusters**	Putative novel variants	Absent	Present

In the Bac6 genome, PHASTER/Phastest identified seven prophage regions. Region 1 (33.7 Kb) was classified as intact and shared high similarity with *Brevibacillus* phage Osiris (NC_028969). Region 2 (48.3 Kb) was also intact, corresponding to *Bacillus* phage vB_BtS_BMBtp14 (NC_048640). Region 3, spanning 42.6 Kb, was categorized as incomplete and showed homology to *Bacillus* phage SPβ (NC_001884). Region 4 (95 Kb) was identified as intact and similarly matched the SPβ phage. Region 5, a 111.4 Kb region, was intact and most like *Bacillus* phage vB_BanS_Tsamsa (NC_023007). Region 6 (39.9 Kb) was also intact and associated with *Bacillus* phage phi105 (NC_004167). Finally, Region 7 (13.1 Kb) was classified as incomplete and showed similarity to *Bacillus* phage AP50 (NC_011523).

As previously reported [15], the pangenome of *B. velezensis* is open, reflecting substantial genomic diversity across strains. A similar pattern was observed for *B. subtilis*, with both datasets showing a large proportion of accessory genes, consistent with the genomic plasticity described for the *B. subtilis* species complex [16]. Although no strictly unique genes were detected exclusively in strains Bac5 (*B. velezensis*) or Bac6 (*B. subtilis*), both strains harbored several low-frequency genes shared with a limited number of genomes, including functions related to carbohydrate metabolism, such as maltose phosphorylase, as well as multiple hypothetical proteins. Gene–trait association analysis using Scoary, performed across all genomes, revealed no statistically significant associations between aquaculture-associated and non-aquaculture strains after multiple testing correction (Benjamini–Hochberg adjusted p > 0.05 for all genes). Although several genes showed nominal significance (uncorrected p < 0.05) and high sensitivity (up to 100%), these associations were not robust after correction and were also present in a subset of non-aquaculture strains.

## 4 Experimental Design, Materials, and Methods

Two bacterial strains were obtained from mangrove sediments samples (pH = 6.1 ± 0.1, Salinity = 24 ± 2, OM = 1.79 ± 0.25) collected in Puerto Hualtaco, Ecuador. The samples were cultured on Chromo agar *Bacillus*, with strains selected based on morphology and relative abundance. High molecular weight DNA was extracted using the Quick-DNA HMW MagBead Kit (Zymo Research, USA), and its quality and concentration were assessed using a Quantus (Promega Inc., USA) and agarose gel electrophoresis. The extracted DNA showed an A260/230 ratio of ∼2.0 and a concentration of approximately ∼30 ng/µL, with electrophoresis revealing high-molecular-weight DNA bands of approximately 1 kb. Whole genome library preparation and sequencing were carried out by Novogene Inc. (Sacramento, USA). Sequencing libraries were prepared using the NEBNext Ultra II DNA Library Prep Kit for Illumina following the manufacturer’s instructions. Libraries with an average insert size of approximately 350 bp were generated and sequenced on the Illumina NovaSeq X Plus platform using paired-end reads of 150 bp (PE150). The sequencing depth targeted approximately 1 Gb of raw data per genome, providing sufficient coverage for draft genome assembly.

Whole-genome assembly of short-read Illumina data was conducted using Bactopia v3.1.0 [17], with assemblies generated through Shovill v1.1.0. Genome quality metrics were evaluated with QUAST v5.3.0 [18], and gene annotation was carried out using Prokka v1.15.6 [19] and the PGAP of NCBI v2025–05–06.build7983. Circular genome visualizations (Fig. 1) were produced with GenoVi v0.2.16 [20]. Taxonomic assignment indicated that both strains belonged to *B. velezensis* (Bac5) and *B. subtilis* (Bac6) based on Kraken v2.0 classification and *16S rRNA* gene similarity determined via BLAST. Average nucleotide identity (ANI) was calculated using fastANI v1.34 [21]. Biosynthetic gene clusters (BGCs) were identified through the BAGEL4 platform and antiSMASH bacterial platform version 8.0.4 [22] with default parameters. Antimicrobial resistance (AMR) genes were detected using the Resistance Gene Identifier (RGI) v6.0.5 with CARD database v4.0.1 [23]. The analysis was performed using the protein homolog model, which detects resistance genes based on curated homology models. Hits were classified according to CARD detection criteria as “Perfect” (100% identity to reference sequences) or “Strict” (high-confidence homologs below perfect identity thresholds). Default parameters implemented in RGI were used. Only “Perfect” and “Strict” hits were considered for downstream analysis.

CRISPR arrays were identified with CRISPRCasFinder v4.2.20 [24,25]. Virulence-associated genes in both strains were identified using ABRicate v1.4.0 against the Virulence Factor Database (VFDB, set A-core dataset), applying minimum thresholds of 70% sequence identity and 60% alignment coverage [26].

Phylogenomic reconstruction (Fig. 2) was carried out through the TYGS platform [27]. Taxonomic nomenclature followed the classifications reported in LPSN (https://lpsn.dsmz.de) [28]. For comparative analysis, the Bac5 and Bac6 genomes were screened against all type-strain genomes available in TYGS using the MASH algorithm [29], and the ten closest genomes for each query were selected. Phylogenomic inference was conducted using the genome BLAST distance phylogeny (GBDP) approach based on the distance formula d5. TBranch lengths are scaled according to GBDP distances, and support values shown at the nodes represent GBDP pseudo-bootstrap values based on 100 replications. The tree was visualized and annotated using iTOL v6 [30].

**Fig. 2:**
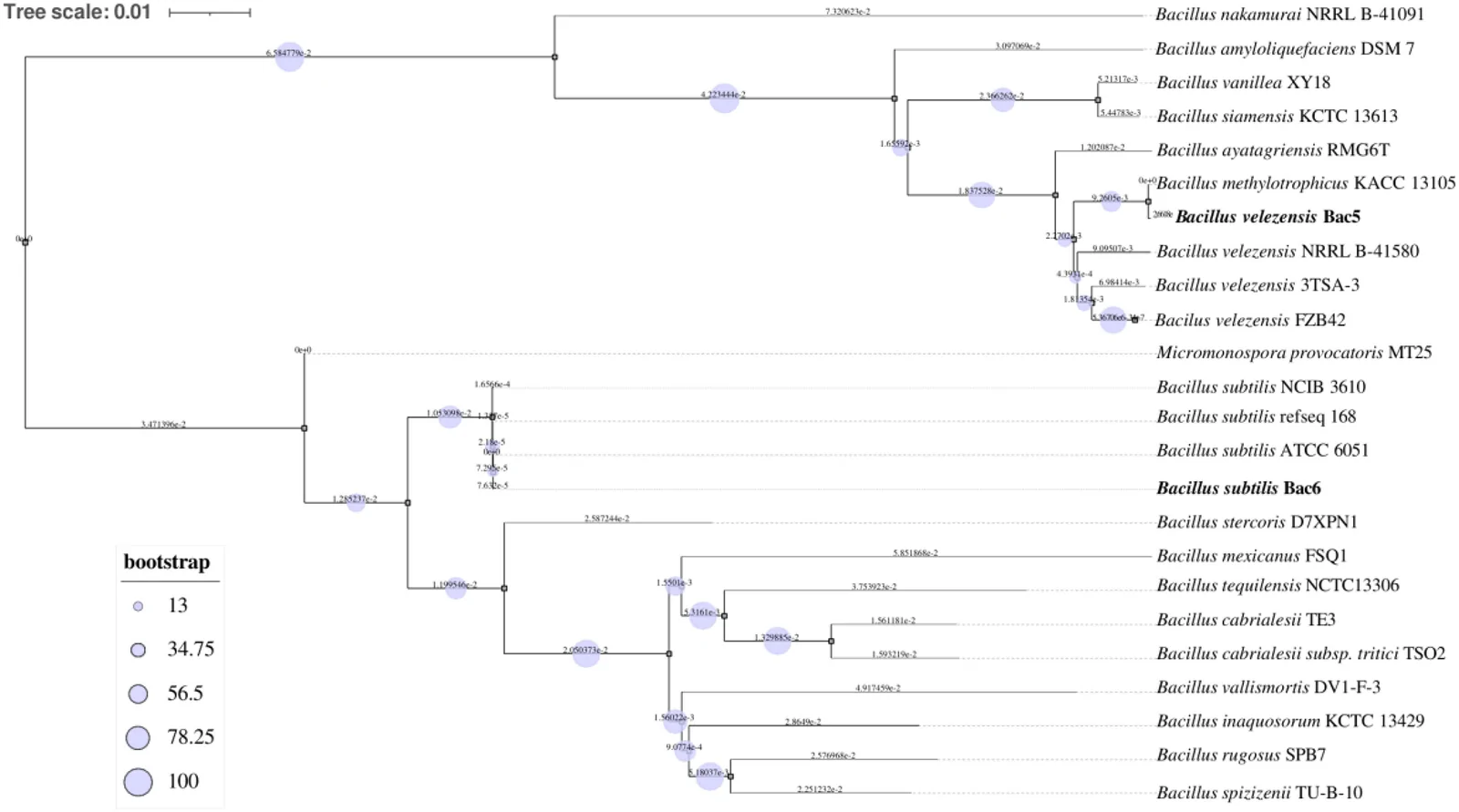
The phylogenetic analysis of *Bacillus* genomes (Bac5 and Bac6 strains) and their closest relatives showed that two isolates grouped within the green (*B. velenzensis*) and yellow (*B. subtilis*) clades. These clusters comprised *Bacillus* species and subspecies with comparable G+C content. Red values above the branches denote evolutionary distances, whereas blue values at the nodes indicate bootstrap support (%).

Genome sequences of *B. velezensis* and *B. subtilis* were re-predicted and re-annotated using Prokka [31]. The resulting GFF3 files were used for pan-genome analysis with the high-speed pipeline Roary [32], applying a 95% amino acid sequence identity threshold (default settings). For comparative purposes, two genome groups were defined. The first group comprised 12 genomes from strains with experimentally validated probiotic activity in aquaculture, as reported in the literature. The second group included 17 genomes randomly selected from strains associated with other applications, mainly in agriculture. The complete list of genomes used in this study is provided in Supplementary Tables 4 and 5. The gene accumulation curve was generated using the Roary output and visualized in R with the ggplot2 package. Genome-wide association analysis was performed using Scoary with default parameters, applying Fisher’s exact test and Benjamini–Hochberg correction to identify genes associated with aquaculture strains [22].

## Limitations

This study reports draft genome sequences of two *Bacillus* strains, and therefore, the assemblies are not completely closed genomes. As a result, some genomic regions may remain unresolved. The use of short-read sequencing generated on the Illumina NovaSeq X Plus platform may limit the resolution of repetitive genomic regions and structural variations, which can lead to fragmented assemblies and the potential omission of repeat-rich regions or plasmid sequences.

In addition, functional annotations and gene predictions were inferred using bioinformatic tools and public databases and therefore represent putative functions that have not yet been experimentally validated. Finally, because the isolates were obtained from environmental mangrove sediments, the findings may be influenced by environmental sampling constraints and may not fully represent the diversity or functional potential of *Bacillus* populations present in other ecological or aquaculture settings.

## Ethics Statement

This research involved bacterial strains and did not include any experiments with humans or animals. The bacterial strains were handled by institutional guidelines and standard microbiological safety practices. Consequently, ethical approval was not required for this study.

## CRediT Author Statement

Guillermo Reyes: Formal analysis, Methodology, Investigation, Data curation, Writing – Original draft, Visualization and Software. Lita Sorroza: Conceptualization, Methodology, Review, Supervision and Funding Acquisition. All authors reviewed and approved the final manuscript.

## Data Availability

SRA-NCBIBacillus velezensis strain:Bac5 Genome sequencing and Bac6 Microbe sample from Bacillus subtilis (Original data)

## Appendix Supplementary materials

Image, application 1

Image, application 2

Image, application 3

Image, application 4

Image, application 5
